# Amino acid insufficiency orchestrates AMPK and mTOR signaling

**DOI:** 10.1016/j.fmre.2026.04.017

**Published:** 2026-04-20

**Authors:** Tianxing Li, Lin Zhao, Da Jia

**Affiliations:** Key Laboratory of Birth Defects and Related Diseases of Women and Children, Department of Paediatrics, West China Second University Hospital, State Key Laboratory of Biotherapy and Collaborative Innovation Center of Biotherapy, Sichuan University, Chengdu 610041, China

## Abstract

**Figure:**
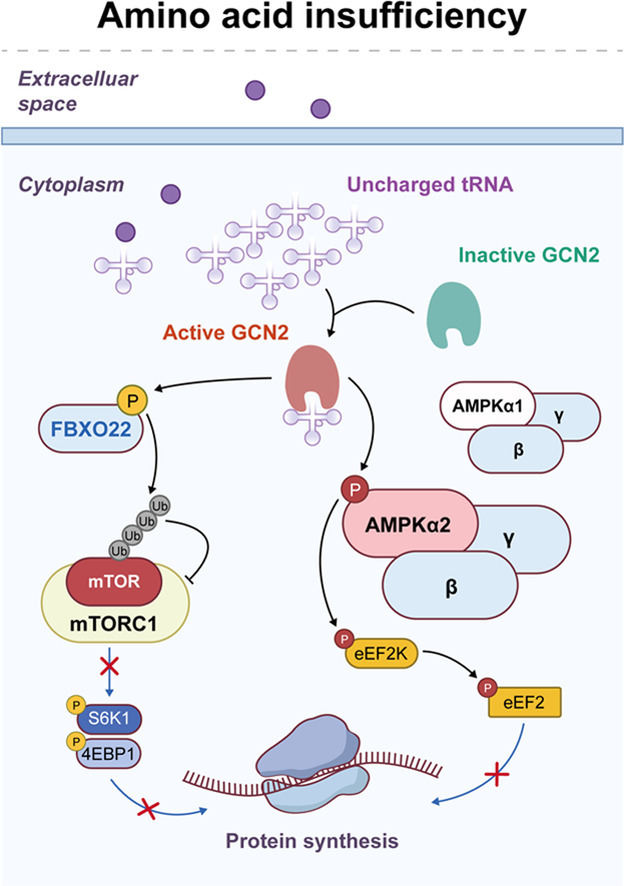

A recent study by Mao et al. in *Cell Metabolism* reported that AMPKα2 specifically responds to amino acid insufficiency to inhibit protein translation, thereby preventing Alzheimer’s disease-like symptoms [1]. Their findings reveal a new regulatory mechanism that allows cells to rapidly halt protein synthesis when amino acids are deficient, coordinating with mTOR signaling to determine cellular fate.

Amino acids fuel protein synthesis and modulate metabolism. Under ample supply, mTORC1—a central growth promoter—is switched on. Specific sensors relay the amino acid signal via Rag GTPases, bringing mTORC1 to lysosomal membranes to initiate anabolism [2]. Active mTORC1 then boosts translation by phosphorylating effectors, including S6K1 and 4EBP1 [3]. However, amino acid deficiency can disrupt this system, employing ubiquitination as a primary shut-off mechanism [4]. The cascade starts with uncharged tRNA engaging GCN2, which directs FBXO22 to tag mTOR with ubiquitin (Fig. 1 left). This tag directly dials down mTOR’s activity, putting protein synthesis on hold [4].

**Fig. 1:**
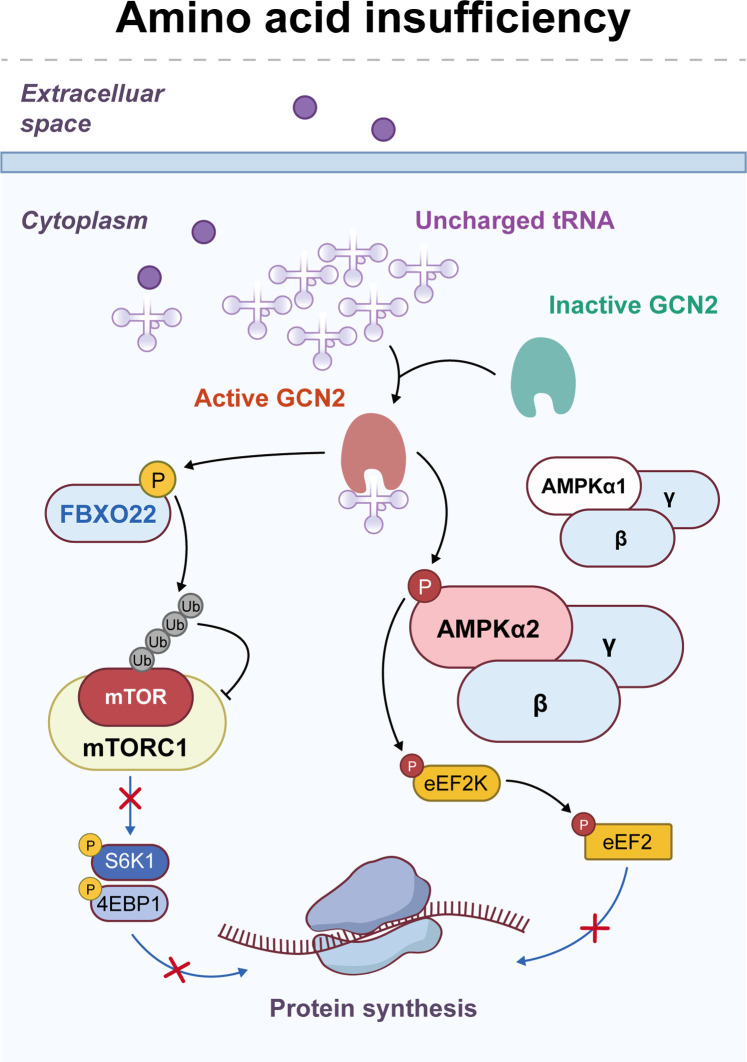
**Cooperative Inhibition of Protein Synthesis by AMPK and mTOR under Amino Acid Insufficiency.** Amino acid insufficiency causes uncharged tRNA to accumulate, and this accumulation then activates GCN2. Left: Activated GCN2 phosphorylates FBXO22. After that, FBXO22 triggers the ubiquitination of mTOR, which suppresses mTOR’s function. This suppression reduces the phosphorylation of S6K1 and 4EBP1, ultimately leading to the inhibition of protein synthesis. Right: When GCN2 is activated, it specifically phosphorylates and activates AMPKα2, but not AMPKα1. This activation triggers a phosphorylation cascade through eEF2K, which inactivates eEF2. When eEF2 is inactivated, peptide elongation is arrested, and this leads to the inhibition of protein synthesis.

The AMPK complex, a key metabolic regulator, contains catalytic α1 and α2 subunits. Their kinase domains are highly similar, granting equivalent ability to phosphorylate common targets [5]. Their primary divergence lies in tissue localization: α1 is abundant in liver, adipose, and kidney tissues, while α2 is chiefly found in skeletal muscle, heart, and neurons [6]. Within neurons, AMPK-mediated translation arrest is a critical survival response. Given its strong neuronal presence, α2 likely contributes more significantly than α1 to neuronal stability.

Traditionally, AMPK has been recognized as a vital detector of metabolic stress cues, such as calcium flux, AMP/ATP ratio, and fructose-1,6-bisphosphate (FBP) [7]. When activated, AMPK sets off a phosphorylation chain reaction via eEF2K, which shuts down eEF2. This shutdown stops eEF2 from helping ribosomes move, quickly putting a pause on peptide chain elongation [8]. Given the exceptionally high energy demands and inherent vulnerability of neurons, this mechanism is far more crucial here than in other tissues. However, whether AMPK can directly perceive amino acid levels and thereby regulate protein synthesis during amino acid deficiency remains a question requiring further investigation. Mao et al. found that AMPKα2, but not AMPKα1, is directly activated by amino acid insufficiency to inhibit protein synthesis (Fig. 1 right). The absence of AMPKα2 leads to the buildup of tau and β-amyloid (Aβ) aggregates in neuronal cells, which ultimately results in apoptosis. These results establish a new function for AMPK in sensing nutrient stress. They also point to AMPK’s possible role in counteracting the progression of neurodegenerative diseases.

Alzheimer’s disease (AD) is driven by the accumulation of pathological tau and Aβ proteins, which form toxic aggregates in the brain [9]. Essentially, the disease can be viewed as a disorder caused by an imbalance in the proteostasis network, wherein the formation of pathological tau and Aβ aggregates directly results from protein misfolding, abnormal aggregation, and impaired clearance [10]. Furthermore, AMPK is inactive in AD, and this impairment is linked to aberrant tau phosphorylation [11]. Mao et al. observed that in AD assays, AMPKα2^−/−^ mice, but not AMPKα1^−/−^ mice, experienced more severe cognitive and behavioral decline than WT mice [1]. Correspondingly, mice with a genetic modification that makes AMPKα2 unable to be phosphorylated (the T172A mutant) developed the same AD-like traits as AMPKα2^−/−^ mice. These findings demonstrate that losing phosphorylated AMPKα2 (at T172) in mice directly drives AD-like phenotypes, indicating that AMPKα2’s role in curbing protein translation is critical for guarding against AD development.

Mechanistically, the authors identified GCN2 as the kinase that phosphorylates AMPKα2 at T172 [1]. Previous studies have reported that under amino acid deficiency, uncharged tRNA activates GCN2, thereby triggering the eIF2α-ATF4 axis [12]. The authors’ newly identified AMPKα2 axis provides another critical functional endpoint for this signaling cascade. Once active, GCN2 then adds a phosphate group to AMPKα2 at the T172 site (Fig. 1 right). Moreover, the loss of GCN2 in neuronal cells concurrently induced the aggregation of tau and Aβ and triggered apoptosis. Notably, GCN2^−/−^ mice developed AD-like traits that were similar to those seen in AMPKα2^−/−^ mice.

While Mao et al.’s study focuses on mouse models, it’s supported by steady evidence from human pathology work [1]. The study included a cohort of 40 AD patients and 40 matched healthy controls, providing initial insights despite the sample size being modest for definitive clinical validation. AD patients’ white blood cells had significantly less phosphorylation of AMPKα-T172 and GCN2-T899. This molecular trait lined up with a distinct metabolic profile, total protein was higher in plasma and leukocytes, and plasma levels of 13 proteinogenic amino acids were lower.

Finally, another notable finding of the study was that AMPK activation, as well as restriction of branched-chain amino acids (BCAAs) or dietary protein, protects against AD-like symptoms in mice in an AMPKα2-p-T172-dependent manner [1]. The classic oral hypoglycemic drug metformin exerts its physiological effects through multiple mechanisms to activate AMPK: on the one hand, it inhibits mitochondrial complex I to increase the AMP/ATP and ADP/ATP ratios in high doses; on the other hand, it promotes AMPK phosphorylation and activation via the lysosomal pathway in low doses [13]. The concentration of metformin used in Mao et al.’s study was insufficient to activate mitochondrial AMPK, suggesting that the GCN2-AMPKα2 axis relies more on the lysosomal pathway. Considering metformin’s widespread clinical use as an AMPK activator [14], Mao et al.’s findings thereby provide a mechanistic basis for the use of metformin in AD prevention and open a potentially therapeutic avenue for nutritional interventions.

In conclusion, Mao et al.’s study demonstrates how AMPKα2 specifically senses amino acid shortages and uses this signal to inhibit protein translation, which indicates that AMPKα possesses a dual-sensing capability—it is responsive to both energy signals and amino acid signals, thereby serving as a regulatory hub that integrates nutrient and environmental signals. This finding also highlights a key protective role of this pathway in fighting AD. The uncharged tRNA-GCN2-AMPKα2 axis directly suppresses protein synthesis, thereby blocking the accumulation of pathological tau and Aβ proteins at their source to prevent AD. Their work further explains that AMPKα1 and AMPKα2 split their tasks: they can detect and combine different metabolic signals, then coordinate the cell’s adaptive responses to keep its internal environment stable. This work not only deepens our mechanistic understanding of how AMPKα responds to metabolic signals but also provides a promising way for the early prevention of AD. Furthermore, the application of limiting BCAAs or dietary protein offers novel potential therapeutic strategies for diseases beyond AD that are caused by aberrant protein synthesis, such as cancer.

Despite offering a novel perspective on AMPK and AD pathogenesis, it should be noted that the study by Mao et al. has inherent limitations. Furthermore, given that amino acid availability is already reduced in patients with AD, further dietary restriction of amino acids may not be beneficial and could even exacerbate underlying metabolic imbalances. Therefore, caution is warranted when considering the translation of such nutritional interventions into clinical practice. Meanwhile, the human data in the study were derived from peripheral blood cells and plasma rather than specific brain regions; the role of the GCN2-AMPKα2 axis within specific brain areas and across distinct neuronal populations remains to be investigated. Lastly, while the authors focused on the inhibition of protein synthesis via activation of the GCN2-AMPK axis to prevent AD, the GCN2-ATF4 axis and the AMPK-ULK1-autophagy axis are equally important in AD, as they respectively suppress protein synthesis and facilitate the clearance of accumulated proteins.

Like many groundbreaking studies, Mao et al.’s study raises a host of questions. Firstly, upon sensing uncharged tRNA during amino acid deficiency, GCN2 is known not only to activate AMPKα2 but also to mediate the ubiquitination of mTOR [4], both of which ultimately suppress protein synthesis. We are then prompted to ask: how are these two signaling branches coordinated? Do they act simultaneously or sequentially? Considering that GCN2 phosphorylates AMPKα2 through a one-step direct mechanism, whereas inducing mTOR ubiquitination requires a multi-step cascade involving phosphorylation, translocation, and ubiquitination, the activation of AMPKα2 by GCN2 likely occurs earlier than the ubiquitination event. Secondly, AMPK is traditionally known to be activated by energy stress and glucose deprivation. This study reports its activation in response to amino acid deficiency. Some key questions emerge: what is the full scope of its sensory capacity? Long-chain fatty acyl-CoA has been reported to directly bind and activate AMPK [15], demonstrating that AMPK also functions as a direct sensor for multiple nutrients. Hence, it is crucial to discover more nutrients that can directly activate AMPK. Finally, there is a crucial question about how widely this mechanism applies: Does the way AMPK detects amino acids work the same across all different non-neuronal tissues? If so, this discovery would enable a novel therapeutic strategy for conditions characterized by dysregulated protein metabolism, and cancer stands as a foremost example for its application.

## CRediT authorship contribution statement

**Tianxing Li:** Writing – original draft. **Lin Zhao:** Writing – review & editing. **Da Jia:** Writing – review & editing.

## Declaration of competing interest

The authors declare that they have no conflicts of interest in this work.
